# Effect of active immunization against GnRH on testosterone concentration, libido and sperm quality in mature AI boars

**DOI:** 10.1186/1751-0147-54-33

**Published:** 2012-05-28

**Authors:** Ronaldas Bilskis, Neringa Sutkeviciene, Vita Riskeviciene, Aloyzas Januskauskas, Henrikas Zilinskas

**Affiliations:** 1 Veterinary Academy of Lithuanian University of Health Sciences, Tilzes str. 18, LT-47181, Kaunas, Lithuania

**Keywords:** Testosterone, Sperm quality, Immunization against GnRH, Boars.

## Abstract

**Background:**

The aim of the present study was to investigate the efficacy of the Improvac on testosterone concentration in blood serum, sexual behavior and sperm quality in matured AI boars. A total of nine Danish Landrace AI boars were included in the analysis.

**Methods:**

The trial period lasted for 15 weeks and was divided into four periods: Control period: three weeks before vaccination; Period I – four weeks after first vaccination; Period II – four weeks after second vaccination, Period III – four weeks after third vaccination. Blood and sperm samples were collected at weekly intervals. Freshly collected sperm samples were analyzed.

**Results:**

Testosterone concentration correlated with libido (r = 0.531; p < 0.001), volume of ejaculate (r = 0.324; p < 0.001) and the percentage of morphologically normal spermatozoa (r = 0.207; p < 0.05). Testosterone concentration rised significantly (p < 0.05) in 5–6 week of trial, e. i. after the first dose of Improvac and after this peak the level of testosterone further progressively decreased (p < 0.05).

**Conclusions:**

Results from this study indicate that active immunization of sexually matured boars against GnRH has negative impact on testosterone concentration, sexual behavior, volume of ejaculate and total number of normal spermatozoa in ejaculate.

## Background

Surgical male pig castrations are frequently performed as an aid to control an unpleasant taste and flavor of the meat, so-called boar taint, exhibited by entire male pigs [1]. Compared to castrates, entire male pigs have a better growth performance [2], feed conversation ratio [3-7] and higher lean meat percentage [3,8,9]. In some countries, culled AI boars are being castrated, slaughtered and their meat used to prepare meat products. Surgical castration of these boars, although with anesthesia, is still complicated because of post-operative infections, stress, lost weight and etc. A more practical and animal-friendly alternatives to surgical castration would be the production of entire male pigs, or immunization against GnRH, known as immunocastration [10,11].

Gonadotropin-releasing hormone (GnRH) is a decapeptide hypothalamic hormone that plays a central role in the regulation of mammalian reproduction. GnRH selectively stimulates the release of luteinizing hormone (LH) and follicle stimulating hormone (FSH) from the anterior pituitary to promote maturation of ovarian follicles or spermatogenesis [12,13]. The technique of immunocastration is based on active immunization in order to produce antibodies against GnRH. The GnRH vaccine stimulates antibody production to inactivate endogenous GnRH, and thereby reduces release of gonadotropin hormones leading to temporary gonadal atrophy [14], and as a result, regression of the reproductive organs, and “immunological castration” [15]. The manufacturer of the vaccine recommends using two doses that are given at least 4 weeks apart with the second dose given 4–6 weeks prior to slaughter. An anamnestic immunological response occurs resulting in high levels of antibody against GnRH 10–14 days after second dose, as described by the manufacturer. In a present study three doses of vaccine were used to monitor effects of prolonged administration of vaccine on adult animals that had pervious history of semen production for AI.

Active immunization against gonadotropin releasing hormone (GnRH) disrupts the hypothalamic-pituitary-gonad axis, thereby inhibiting the function of the Leydig cells and delaying/suppressing the attainment of sexual development of testis and steroid synthesis [16]. Immunization against GnRH reduces the concentrations of testicular hormones testosterone, the testicular size and weight as well as the diameter of tubuli seminiferi of testes, sperms number and aggressive behavior in young [16-19] and adult boars [20]. Limited number of studies evaluated the effectiveness of immunocastration in adult boars once they have completed their productive live in the breeding farm and should be castrated before slaughtering. The present study was designed to investigate the efficacy of the Improvac applied as double or triple injections on testosterone concentration, libido and sperm quality in culled AI boars.

## Methods

### Animals and experimental design

A total of nine Danish Landrace mature AI boars were included in the analysis. All boars investigated were clinically healthy AI boars that were culled from commercial AI station due to rotation program of an AI station. Before inclusion to the trial all boars were used for semen production for AI. The mean age of animals were 34.9 ± 10.7 months. All boars were kept in separate pens. Water was available ad libitum. All boars were fed commercial boar diet [21]. All boars were vaccinated with Improvac® (Pfizer Ltd). Vaccination was performed as per manufacturer’s instructions (2 mL/pig injected subcutaneously just behind and below the base of the ear). Animals were observed daily for general health. The trial period lasted for 15 weeks and was divided into the four periods. Control period – three weeks before vaccination; Period I – four week period between vaccination I and II; Period II – four week period between vaccination II and III, Period III – four weeks after third vaccination. Blood and sperm samples were collected at weekly intervals.

### Blood analysis

Blood samples for analysis of testosterone were taken after every sperm sampling at 9.00-10.00 a.m. from the ear vein into plain glass tubes. The samples were transported to the laboratory within ½ – 1 hour. Blood was centrifuged at 3000 rpm for 5 min and 2 ml of the serum were transferred to Eppendorf test tube, using 1 ml Paster pipette (Einweg-Pasteurpipetten, Carl Roth GmbH, Germany). The tubes were immediately frozen and stored at −20 °C until the analysis. Testosterone concentrations (ng/mL) were analyzed by a computerized Multi-Detection Microplate Reader Synergy^TM^ HT (Bio-Tek® Instruments, Inc., USA, 2004) with the DIAsource TESTO-EASIA Kit (DIAsourse ImmunoAssays S.A., Belgium) according to the manufacturer’s instructions.

### Sexual behavior evaluation

Libido levels were assessed according to Ren et al. [22] by a 10 points score system in 9 boars without natural mating experience. The libido level of each boar was a mean value from three continual evaluations during the training period. If the boar showed optimal sexual behavior (including champing of jaws producing saliva, sniffing the anal-genital region or head of dummy sow, nudging or noising the flanks of dummy sow and mounting, optimal reaction time (less than 2 min) and duration of ejaculation (no les 7 min) it was scored 8 point. The hyper sexual behavior was scored 10 points. If the boar refused to mount the dummy sow during three evaluation times it was scored zero point and was classified as lacking libido.

### Semen analysis

Boar ejaculates were collected by the gloved-hand technique. The volume of each ejaculate (ml) was recorded and freshly ejaculated semen was extended in the BTS/Androhep (v/v) extender according to the routine AI centre practice. Extended semen was used for analysis. Motility of spermatozoa was examined subjectively at 37^o^ C under phase-contrast microscope Olympus BH2 with a pre-warmed 37 °C stage (Olympus Optical Co., Ltd., Japan) using a 100 × magnification. Motility was analyzed on 5-μl aliquots of fresh semen.

Sperm tail defects were determined in wet preparations (an aliquot of semen was fixed in buffered formol-saline solution [23] under the phase-contrast microscope at 400 × magnification. Sperm head defects were determined in dry preparations stained according to Williams and Savage [24]. The total amount of pathologic spermatozoa were classified: Tail defects (sperm tail defects, abnormal midpieces and the incidences of tail abnormalities); Head defects (pear shape, narrow at base, abnormal contour, undeveloped, loose abnormal head, narrow, big, little normal, short – broad) and Other defects (spermatozoa with proximal and distal cytoplasmic droplets). Sperm concentration was measured in a Bürker cell counting chamber.

### Statistical analysis

Statistical analysis was performed using the *SPSS* statistical package *No. 15* for Windows (SPSS for Windows 9.0, SPSS Inc., Chicago, IL, USA, 1989–1995). Data included in the model were analyzed using descriptive statistics (means ± SD) and one-way ANOVA analysis. Differences among investigated periods were analyzed by LSD method (α = 5 %). The data was considered to be statistically significant when: * p < 0.05; ** p < 0.01; *** p < 0.001. Correlation among dependent variables and strength of the direct relation was evaluated by Pearson’s correlation coefficients.

## Results

All animals well tolerated vaccine injection and remained in good general health during the entire experiment. The testosterone concentration, libido, percentage of motile spermatozoa, volume of ejaculate, sperm concentration in the ejaculate and the total amount of the abnormal spermatozoa of the experimental boars during periods are presented in Table1.

**Table 1:** Blood serum testosterone concentrations and sperm quality and quantity parameters during the trial

Variables	Periods			
	Control^a^	I^b^	II^c^	III^d^
Testosterone, ng/ml	3.47 ± 1.62^b,c,d^	5.05 ± 2.34^a,c,d^	1.35 ± 1.02^a,b,c^	0.25 ± 0.17^a,b^
Libido, points	8.00 ± 0.00^b,c,d^	10.00 ± 0.0^a,c,d^	8.65 ± 0.56^a,b,d^	5.00 ± 2.04^a,b,c^
Sperm motility, %	55.74 ± 12.07	51.67 ± 14.24	56.94 ± 8.89^d^	48.33 ± 15.72^c^
Volume of ejaculate, ml	190.19 ± 84.69^c,d^	192.06 ± 56.65^c,d^	156.11 ± 51.25^a,b,d^	105.17 ± 34.75^a,b,c^
Concentration, 10^9^ejaculate	19.65 ± 9.27^c^	23.70 ± 8.77	27.67 ± 16.35^a^	24.57 ± 10.37
Total amount of abnormal spermatozoa	35.41 ± 26.24^d^	32.67 ± 24.28^d^	42.17 ± 23.55^d^	73.29 ± 26.01^a,b,c^

^a, b, c, d,^ Means with different superscript in columns are significantly different (p < 0.05). Data presented as mean ± SD.

**Figure 1:**
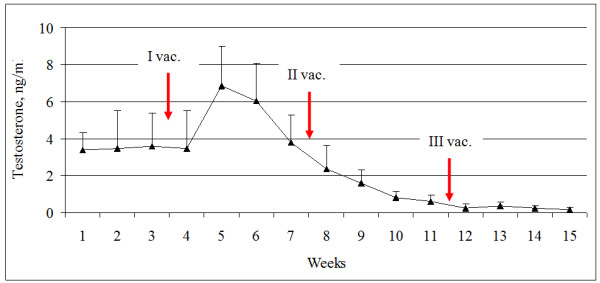
Dynamics of testosterone concentration during the entire experimental period. N = 9 animals. Arrows indicate the vaccination dates.

The testosterone concentration was steady in control group, but rised significantly in all animals after the first vaccination (p < 0.05) injection. Then, after this peak the level of testosterone further progressively decreased. Dynamics of testosterone levels in the blood serum of experimental boars during trial showed in Figure 1.

Testosterone concentration correlated with libido (r = 0.53; p < 0.001) and volume of ejaculate (r = 0.32; p < 0.001), and negatively correlated with percentage of abnormal spermatozoa (r = −0.21; p < 0.05).

In the Control period the libido of the boars was in the optimal level (8.00 ±0.00). The average libido level in boars after first vaccination of Improvac was scored to 10.00 ± 0.00 points of the 10-point scoring system and was considered as hypersexual behavior. In contrast, the libido level after third vaccination was 5.0 ± 2.04 point (p < 0.001). Libido scoring correlated with volume of ejaculate (r = 0.38; p < 0.001) and negatively correlated with total number of spermatozoa having proximal droplets (r = −0.311; p < 0.001), and with the total number of spermatozoa with tails coiled under the head (r = −0.23; p < 0.05) and spermatozoa with abnormal heads contour (r = −0.24; p < 0.05).

The motility of spermatozoa did not differ significantly between the Control and all three trial periods. The difference in sperm motility variables was by 7.4 ± 3.65 % lower in Period III as compared to the Control period (p > 0.05). The percentage of motile spermatozoa correlated negatively significantly with percentage of spermatozoa with proximal (r = −0.25; p < 0.01) and distal (r = −0.25; p < 0.01) droplets, and spermatozoa with simple bent tails (r = −0.41; p < 0.001).

The mean volume of the ejaculate decreased significantly (p < 0.05), but sperm concentration in the ejaculate remained at the same levels (p > 0.05) (Table1).

**Figure 2:**
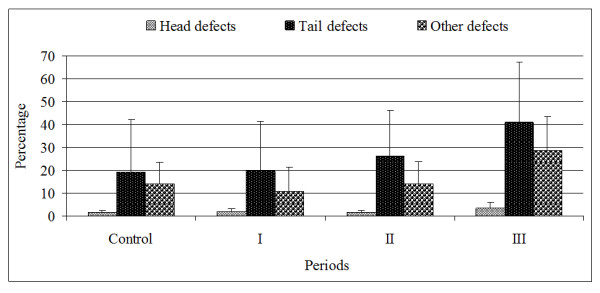
The percentage of spermatozoa head, tails and other defects (spermatozoa with proximal and distal cytoplasmic droplets) during trial periods.

The most common sperm defects detected in ejaculates during experimental periods were spermatozoa with proximal droplets and spermatozoa with simple bent tails. The number of abnormal spermatozoa rose throughout the experiment, but the rise was most prominent in Period III. The lowest percentage of spermatozoa with tail defects was detected at the beginning of the experiment (19.44 ± 22.96 %, Figure 2.). The same trend followed all other defects: tail defects, sperm head defects and other defects.

Our study showed that active immunization against GnRH has a negative impact on reproductive performance of the boars, as it lowers testosterone concentration in boar blood serum, libido, and volume of ejaculate and increases the number of normal spermatozoa in ejaculate.

## Discussion

As far as we are aware, this is the first study to investigate the efficacy of the Improvac in mature (adult) boars and their reproductive performance. The results of other studies showed, that vaccination of young male pigs raised for slaughter was successful when performed twice [16-18]. Also some authors reported that in pig males the effects included reduction of testis weight, reduced plasma testosterone and some histological changes in spermatogenesis and regression in Leydig cell number however, the histochemical analyses suggested that spermatogenesis was disrupted in only few males as a result of the treatment of European wild boar *(Sus scrofa)* with a single dose GnRH vaccine [15]. In our study the vaccine was injected three times because mature/adult and AI boars were used.

GnRH vaccines produce adequate antibody titers are believed to act on the hypothalamus and inhibit synthesis and secretion of GnRH which normally stimulates production of follicle stimulating hormone (FSH) and luteinizing hormone (LH) by the anterior pituitary gland. LH is responsible for initiating testosterone production by the Leydig cells. FSH stimulation of the testes starts the process of the sperm production (spermatogenesis) by initiating spermatogenic epithelium cell division and maturation. In the absence of these hormones, normal stimulation of the testes is compromised and gonadal regression occurs. There were several studies that assessed antibody levels in vaccinated animals [14,15] we did not intend to analyze the variation of the antibody titers, but instead we have assessed the change in the concentration of testosterone before and after immunization with Improvac. Also the boar libido and sperm quality in tested boar were evaluated. The results of our study show that general health was not affected in either of the treated animals, nor did the injection site was show an inflammatory response, indicating that Improvac was well tolerated. This confirms the results of other authors, that no observable site reactions were detected at the time of slaughter on treated adult animals [20].

Successful testicular function depends on the hypothalamic secretion of GnRH, which in turn stimulates LH to act on the testis and initiates production of testicular steroids. Testosterone is synthesized and released from the Leydig cells of the testis, and synthesis of testosterone is dependent on LH stimulation. Park and Yi [25] reported that serum testosterone concentration in adult Duroc and Yorkshire boars 15–22 months of age varied between from 0.73 to 3.06 ng/ml and 2.48 to 5.11 ng/ml respectively among seasons. Ren et al. [22] reported 27.9 ± 22.4 ng/ml the average serum testosterone level from 195 boars aged 300 days. In the Control period of our study the concentration of testosterone in blood serum varied from 0.91 to 6.43 ng/ml. In the present study the average serum testosterone concentrations were similar for all boars at the start of the study. The secretion of testosterone increased in blood serum in week 5 of trial, i.e. after first dose of Improvac (p < 0.05). The rise in testosterone concentration after first vaccination might be a testicular response to disrupted GnRH release due to vaccination. Increase in testosterone production was depicted in increase of sexual behavior as recorded 1 week later. After this peak the level of testosterone further progressively decreased. These observations agree with results obtained in other studies, where testosterone levels decreased after second vaccination in Zamaratskaia’s studies [18] and were lower in immunocastrated males compared with entire males [15]. Low or event undetectable levels of plasma testosterone of immunocastrates up to 8 weeks after the second vaccination indicate a suppression of secretion of testosterone and spermatogenesis. Claus et al. [26] reported that all pigs had a significant increase in antibodies against GnRH from the period before the second immunization and thereafter. The testosterone in their study remained high after the first immunization and decreased 5–10 days after second dose. Claus et al. [27] reported sudden drop of testosterone concentration following second vaccination with the nadir about day 60. Kauffold et al. [28] also found low concentrations of testosterone of up to 0.35 ng/ml in deslorelin treated boars. In support of this interpretation Zanella et al. [29] reported that plasma concentrations of testosterone decreased already 3 h after the injection of GnRH antagonist on thirteen boars older than 1 year of age.

Testosterone concentration correlated positively with boar libido (p < 0.001), volume of ejaculate (p < 0.001), and negatively correlated with percentage of abnormal spermatozoa (p < 0.05). These observations agree with results obtained in studies with large White Duroc × Chinese Erhualian Crossbred boars at three developmental stages, where the testosterone concentration correlated with epididymis weight and libido scores, although libido and serum testosterone level were not correlated with sperm production [22].

Boar libido is affected by genetic, seasonal, social, sexual and psychological factors, thus its intensity is very difficult to measure. The results of our studies showed that the mean of boar libido level after first vaccination of Improvac increased by two points in the 10-point scoring scale. However libido started to decline following second vaccination. This is related to decreased testosterone levels after second and third vaccination. Other studies showed that immunocastration of sexually mature boars eliminate sexual behavior for at least 4 week period [30]. Zamaratskaia‘s studies [18] showed that still 21 weeks after the second vaccination none of the vaccinated boars were able to mount a gilt in estrus.

Sperm motility is an important characteristic in porcine semen assessment [31,32]. Spermatozoa gain motility during ejaculation as pH and bicarbonate concentration increase during mixing of sperm and seminal plasma [33]. Many factors are necessary to produce normal sperm motility: optimal temperature and pH, appropriate levels of reactive oxygen species and adenosine triphosphate (ATP), etc. [34]. However the motility of spermatozoa in the present study revealed no statistically significant difference between the control and three trial periods. Our findings indicated that motility is not directly affected by testosterone concentration, sperm volume and sperm concentration in the ejaculate. These findings are in agreement with other studies performed on pigs [35][36]. Still, normal sperm tail morphology is also necessary for the motion of spermatozoa. Therefore the structural sperm tail abnormalities are responsible for altered/absent sperm motility [37]. The percentage of motile spermatozoa correlated negatively significantly with percentage of spermatozoa with proximal (p < 0.01) and distal (p < 0.01) droplets, and spermatozoa with simple bent tails (p < 0.001). These results are in agreement with earlier reports [38-41].

The total sperm number per ejaculate was not correlated with the serum testosterone concentrations in this study. Walker et al. [42] in his study evaluating Duroc boars from high and low lines divergently selected over 10 generation for testosterone production in response to a GnRH challenge reported that daily sperm production was not different between lines despite higher serum testosterone concentrations in boars from high lines. This may be because spermatogenesis can proceed at less than maximal testosterone levels. Peter et al. [43] also reported a low correlation between sperm production and the concentration of testosterone in the ejaculate of boars. This indicates that although spermatogenesis requires testosterone, there may be a threshold effect whereby further increases do not result in more sperm production once a certain level of testosterone is achieved [42]. Walker et al. [42] and Ren et al. [22] also reported that sperm production in boars was unaffected under divergent selections for testosterone production and the serum testosterone concentration in mature boars could not be recommended as the indicator for sperms production. Additionally, the total sperm number per ejaculate was not correlated with the libido level, according with previous results [22].

The results of our study showed that the total percentage of abnormal spermatozoa in the ejaculate after second and third vaccination was higher than was in the control period (p < 0.05). The results were expected, because vaccination indirectly locks release of the FSH, and FSH is required for normal spermatogenesis [12,13].

The results presented in this study showed that active immunization against GnRH with Improvac suppressed reproductive function in adult male pigs: evidence by decreased levels of testosterone, boar libido, volume of ejaculate and total number of normal spermatozoa in ejaculate in the immunocastrated AI boars.

## Competing interests

The authors declare that they have no competing interests.

## Authors’ contributions

NS and RB carried out the study, compiled the results and drafted the manuscript. VR participated in statistical analysis of the data and has helped to draft the manuscript. AJ was involved significantly in the study, interpreting data and composing the manuscript. HZ coordinated the study. He has been involved in many of the studies reviewed in this manuscript, and also helped to draft the manuscript. All authors read and approved the final manuscript.
